# 2442: Alcohol reduces the ability to regulate emotion when exposed to evocative partner stimuli in individuals with a history of intimate partner violence

**DOI:** 10.1017/cts.2017.232

**Published:** 2018-05-10

**Authors:** Brandi Fink, Eric D. Claus, James F. Cavanagh, Derek A. Hamilton, Sarah Salway

## Abstract

OBJECTIVES/SPECIFIC AIMS: The objective of this research was to investigate the effect of alcohol and evocative stimuli on heart rate variability (HRV) in partners with a history of intimate partner violence in a placebo-controlled alcohol administration study with an emotion-regulation task. METHODS/STUDY POPULATION: In total, 17 partners (9 females, 8 males) with a history of partner violence participated in a placebo-controlled alcohol administration study with an emotion-regulation task during which HRV measures were collected. In the alcohol condition, participants were administered a mixture of 100 proof vodka and cranberry juice calculated to raise their blood alcohol concentration (BAC) to 0.08%. In the placebo condition, participants consumed a volume of juice equivalent to that consumed in the alcohol condition, but without alcohol. Alcohol and placebo conditions were counter-balanced across participants as were the presentation the blocks of evocative and neutral partner stimuli. RESULTS/ANTICIPATED RESULTS: Controlling for baseline HRV, there was a significant main effect of stimuli (evocative vs. neutral partner stimuli) on HRV in intoxicated partners, *F*_1,16_=16.28, *p*=0.004. There was also a significant main effect of regulation on HRV under conditions acute alcohol intoxication, *F*_1,16_=23.55, *p*=0.001. These effects tell us that intoxicated partners experienced reduced HRV when exposed to evocative stimuli from their partners. These effects also tell us that under acute alcohol intoxication, partners were less able to regulate their emotion when exposed to evocative stimuli than when they consumed a placebo beverage. DISCUSSION/SIGNIFICANCE OF IMPACT: These results suggest that increases in intimate partner violence under acute alcohol intoxication may be the result of reduce HRV. This reduction in HRV would contribute to partners’ inability to response with adaptively in conflict when intoxicated. They also suggest that HRV may be an important target for intervention with partner with a history of intimate partner violence. One method may be Heart Rate Variability Biofeedback which has been shown to increase parasympathetic nervous system functioning, autonomic stability, and emotion regulation.

